# The adaptation of GDL motion recognition system to sport and rehabilitation techniques analysis

**DOI:** 10.1007/s10916-016-0493-6

**Published:** 2016-04-22

**Authors:** Tomasz Hachaj, Marek R. Ogiela

**Affiliations:** Institute of Computer Science and Computer Methods, Pedagogical University of Krakow, 2 Podchorazych Ave, 30-084 Krakow, Poland; Cryptography and Cognitive Informatics Research Group, AGH University of Science and Technology, 30 Mickiewicza Ave, 30-059 Krakow, Poland

**Keywords:** Sport data analysis, Rehabilitation data analysis, Motion capture, Signal classification, Gesture description language

## Abstract

The main novelty of this paper is presenting the adaptation of Gesture Description Language (GDL) methodology to sport and rehabilitation data analysis and classification. In this paper we showed that Lua language can be successfully used for adaptation of the GDL classifier to those tasks. The newly applied scripting language allows easily extension and integration of classifier with other software technologies and applications. The obtained execution speed allows using the methodology in the real-time motion capture data processing where capturing frequency differs from 100 Hz to even 500 Hz depending on number of features or classes to be calculated and recognized. Due to this fact the proposed methodology can be used to the high-end motion capture system. We anticipate that using novel, efficient and effective method will highly help both sport trainers and physiotherapist in they practice. The proposed approach can be directly applied to motion capture data kinematics analysis (evaluation of motion without regard to the forces that cause that motion). The ability to apply pattern recognition methods for GDL description can be utilized in virtual reality environment and used for sport training or rehabilitation treatment.

## Introduction

Motion capture (MoCap) is a powerful technology with many possible applications. The dimensionality of output signal stream from MoCap system depends on number and type of sensors or tracked body joints in virtual skeleton that are used [1–4]. Mostly often each body joint has three or six degrees of freedom. Three are linear coordinates in Cartesian frame with versors (x, y, z) and other three are angles that define the orientation of the body segments. Those values are not used directly by the system but rather features values are calculated as derivatives of original data. The features selection and extraction methods are for example Gabor [5] or Haar filters [6]. Dimensionality reduction can be done with principal components analysis (PCA) [7] or other approaches [8]. The movement representation is often invariant under rigid transformation [8] and can be for example angular representation of the skeleton joints [9] where each pose is described using an angular representation of the skeleton joints.

Many methods have been yet proposed for human actions and movements evaluation and recognition. That type of analysis is important for calculation of biomechanics parameters of actions, for evaluation ones activates and lifestyle or during rehabilitation [10, 11]. Among proposed methods that can be used for signals and action recognition are approaches that are often used for signal identification and pattern recognition. The most popular are Hidden Markov Models (HMM) [10, 12, 13], support vector machines (SVM) [5, 9, 14], decision forests [9, 15], Gaussian process dynamical models [16], K-means clustering [6], nearest neighbor classifier [17], Bayes classifier [18], dynamic Bayesian networks [19], syntactic method [6, 20, 21] and rule based methods – for example Gesture Description Language (GDL) [22–24]. GDL classifier uses a rule – based approach with memory stack. The memory stack holds the captured MoCap data frames, features and classes to which a sequence of MoCap frames are classified. GDL method uses specially designed scripts that hold the definition of features calculated from MoCap input stream. Those features are used to design rules that have if-else form and define the key frames of actions. Key frames are ordered in sequences. If the sequence of key frames appears in memory stack in a given time restriction the ongoing action is classified to a given class. This approach is somehow similar to HMM classifier.

The wide comparison of GDL methodology to other recognition system is discussed in other papers [25–27]. This comparison includes most important aspects like comparison of action description methodology, geometric interpretation of those descriptions, training algorithm and applications. The example results for various physical activates is presented in papers [22–26, 28–31] and includes common-life actions, gym exercises and Oyama and Shorin-Ryu karate techniques.

So far the GDL was used mainly for classification of MoCap data stream from multimedia devices (for example Microsoft Kinect) however we need to create a unified approach that would enable not only classification but also analysis of MoCap signals from high-end hardware. That type of devices is often used to support spot coaches in training optimization and physicians in rehabilitation process [10, 11, 32]. The requirements for this new approach is that it has to be capable to create complex features definitions (that could be for example used for kinematic analysis) and have to be fast enough for real-time data analysis (performance is very important aspect of every medical system [33]). The main novelty of this paper is presenting the adaptation of Gesture Description Language (GDL) methodology to sport and rehabilitation data analysis and classification. In the following sections we will present, evaluate and discuss proposition of such methodology.

## Materials and methods

In this section we will present the novel adaptation of GDL methodology to analysis and classification of MoCap data for sport and rehabilitation applications. To do so we need to enhance the possibilities of features definition of scripting language that is inherent part of GDL classifier. We did it by replacing the old GDLs scripting language with Lua.

### Lua application in GDL paradigm

Lua is a dynamically typed language that can be easily integrated with other computer languages and applications. Due its simplicity and easily extensions it is a popular scripting technology for other computer systems. Lua is used in scientific computing like linear algebra, neural networks, numeric optimization routines and many more [34–36]. Lua was also used in an aspect-oriented infrastructure to handle dynamic programming tasks [37]. Lua is also used in middleware design and development [38, 39] also in robotics and embedded systems [40–43]. This programming language is very popular tool for writing high-level scripts for other computer systems [44–46]. In paper [47] authors discuss what mechanisms Lua features to achieve its flexibility and how programmers use them for different paradigms.

**Fig. 1:**
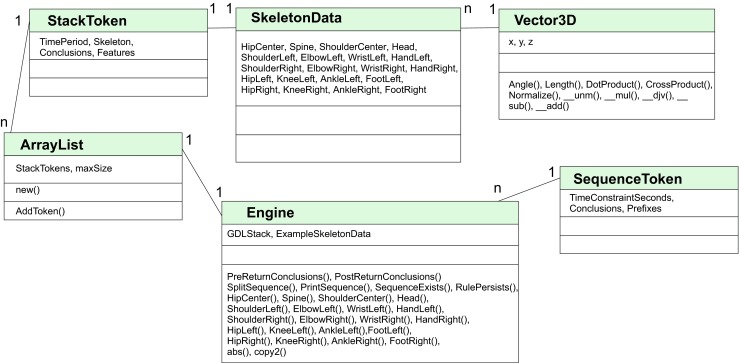
This figure presents a class diagram for Lua implementation of GDL classifier


The proposed Lua implementation is based on Lua 5.2 and JAVA hosting application. Lua functions are called by LuaJ library. GDL engine uses five classes and one script file Engine.lua with global variables and functions - see a class diagram presented in Fig. 1. A very basic script that can be used to detect situation when right hand is under head might looks as follow:

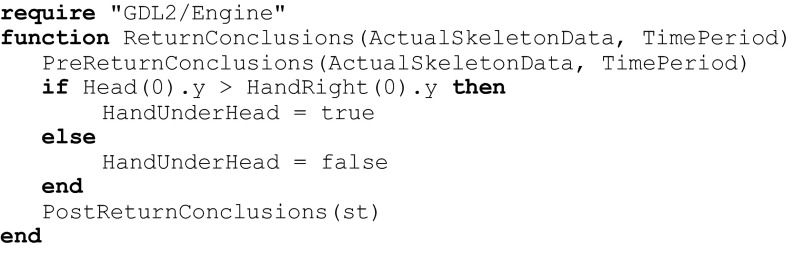


The ReturnConclusions function is called by hosting application to pass the tracking parameters. The proposed Lua – based framework can be easily adapted to anybody joints set simply by configuration of joints definition in SkeletonData class and functions in Engine.lua.

### Features calculation

The adapted GDL classifier uses standard Lua syntax to define features. There are three types of features: logical, numeric and vector. The logical feature role is identical to conclusion from GDL specification and it is represented by ‘boolean’ data type in Lua. Only those logical values that equals ‘true’ are passed to hosting application. The numeric data type has a floating-point value and it is represented by ‘number’ data type in Lua. The vector data type has three floating-point values and is represented by user-defined class Vector3D. There are also definition of most important vectors operation like vector sum, multiplication by a number, dot product, cross product etc. that can be usable for kinematics or kinetics analysis [48] (the basic trigonometric functions like sinus are already supported by Lua).

**Fig. 2:**
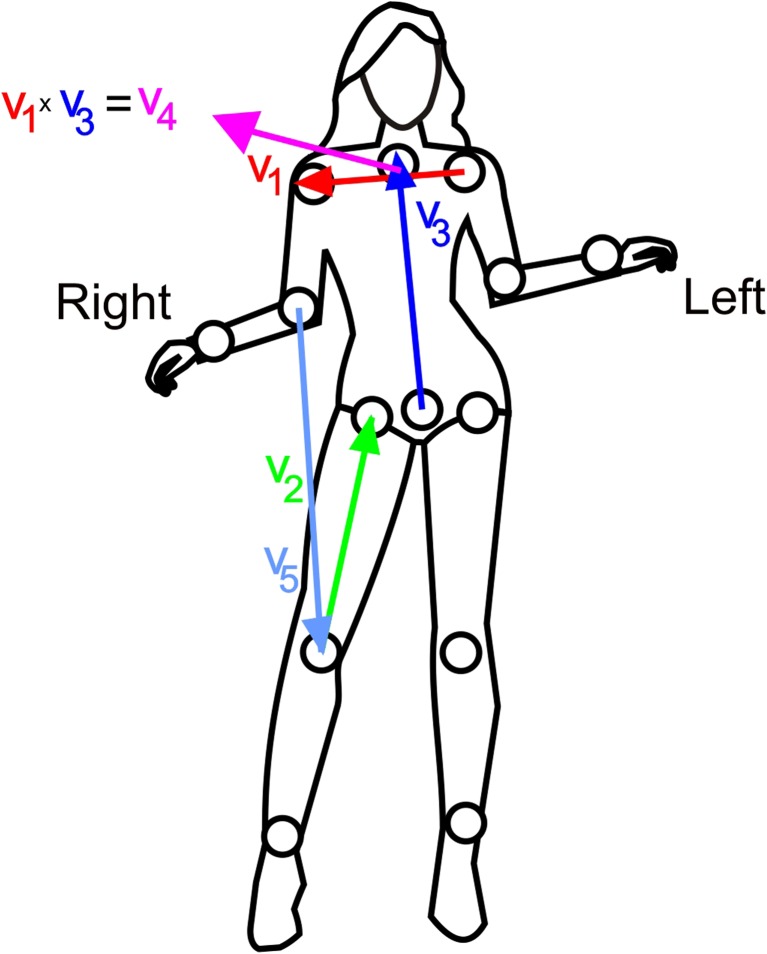
This figure presents vectors set that was used to generate example features

In Fig. 2 we present vectors set that was used to generate features set that was used for recognition hiza-geri karate kick. We have defined six angle-based features:

1 $$ \left\{\begin{array}{c}\hfill {A}_1=\measuredangle \left({v}_1,{v}_2\right)\ \hfill \\ {}\hfill {A}_2=\measuredangle \left({v}_3,{v}_2\right)\hfill \\ {}\hfill {A}_3=\measuredangle \left({v}_4,{v}_2\right)\hfill \\ {}\hfill {A}_4=\measuredangle \left({v}_1,{v}_5\right)\hfill \\ {}\hfill {A}_5=\measuredangle \left({v}_3,{v}_5\right)\hfill \\ {}\hfill {A}_6=\measuredangle \left({v}_4,{v}_5\right)\hfill \end{array}\right. $$

Where ${A}_1,\dots, {A}_6$ are angles calculated between vectors ${v}_1,\dots, {v}_5$ visualized in Fig. 2. Vectors are defined by tracked body joints.

The Lua implementation looks as follows:
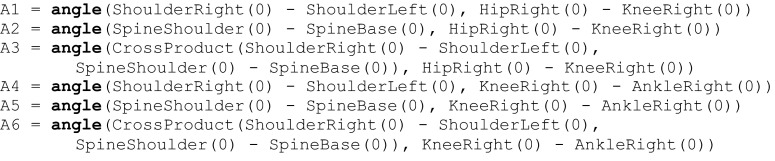


Where angle is a function that finds angle between two vectors on the plane designated by those vectors.

**Fig. 3:**
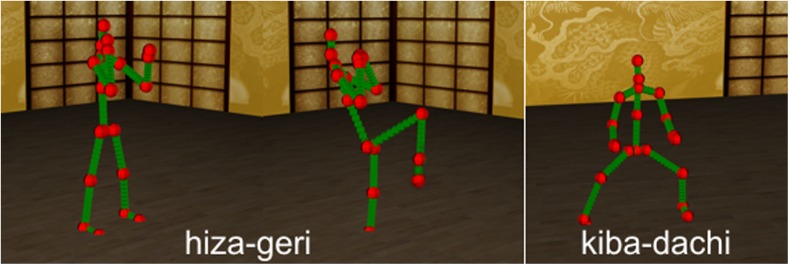
This figure presents important phases of karate actions: Hiza-Geri kick and Kiba-Dachi stance. The Mo-Cap data is visualized in 3D virtual environment

In Fig. 3 we present 3D visualizations of important phases of selected karate actions we used in evaluation of our methodology.

**Fig. 4:**
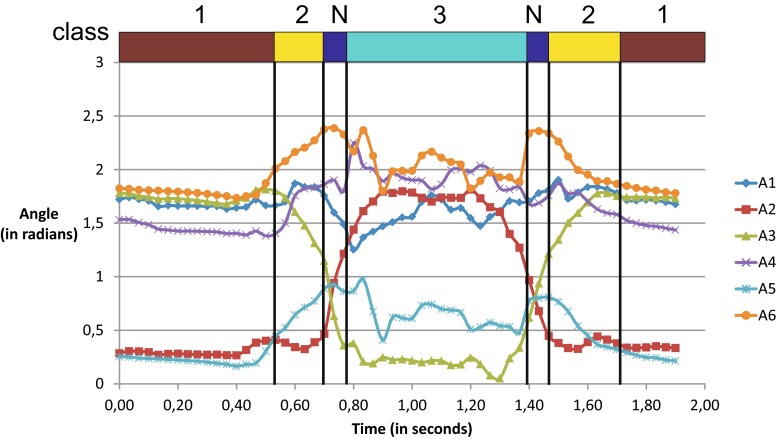
This figure presents features time series generated for single Hiza-Geri kick recording. The *horizontal axis* represents time and the vertical axis the angle. Each time series stands for one of the feature from (1). On the top of the plot there are *color bars* that indicate to which GDL key frame the signal sample has been classified. Color codes are the same as in Fig. 5. Number 1, 2 and 3 are key frames numbers (there are totally three key frames in this particular Hiza-Geri definition). Symbol N represents the time sample in which signals have not been classified to any key frame

In Fig. 4 we present plot of features values defined by (1) for a recording of single Hiza-Geri kick done with Kinect 2 depth camera. Above the plot are horizontal bars with color-coded information about key frames to which current frame was assigned by GDL classifier. Brown is the first key frame, yellow the second, cyan the third. Blue stands for lack of assignment (N – not assigned).

### Pattern recognition with GDL approach

The recognition process of action pattern in adapted implementation is mainly the same as in [25]. The only difference is a reasoning module. We have found that in the previous implementations users seldom used some features of it and might even not be aware of its existence. Due to this fact hardly ever users design GDLs that reference to rule conclusion before it is defined in next rule. Also those types of constructions are not required in automatic training algorithm (R-GDL) about which we write below.

**Fig. 5:**
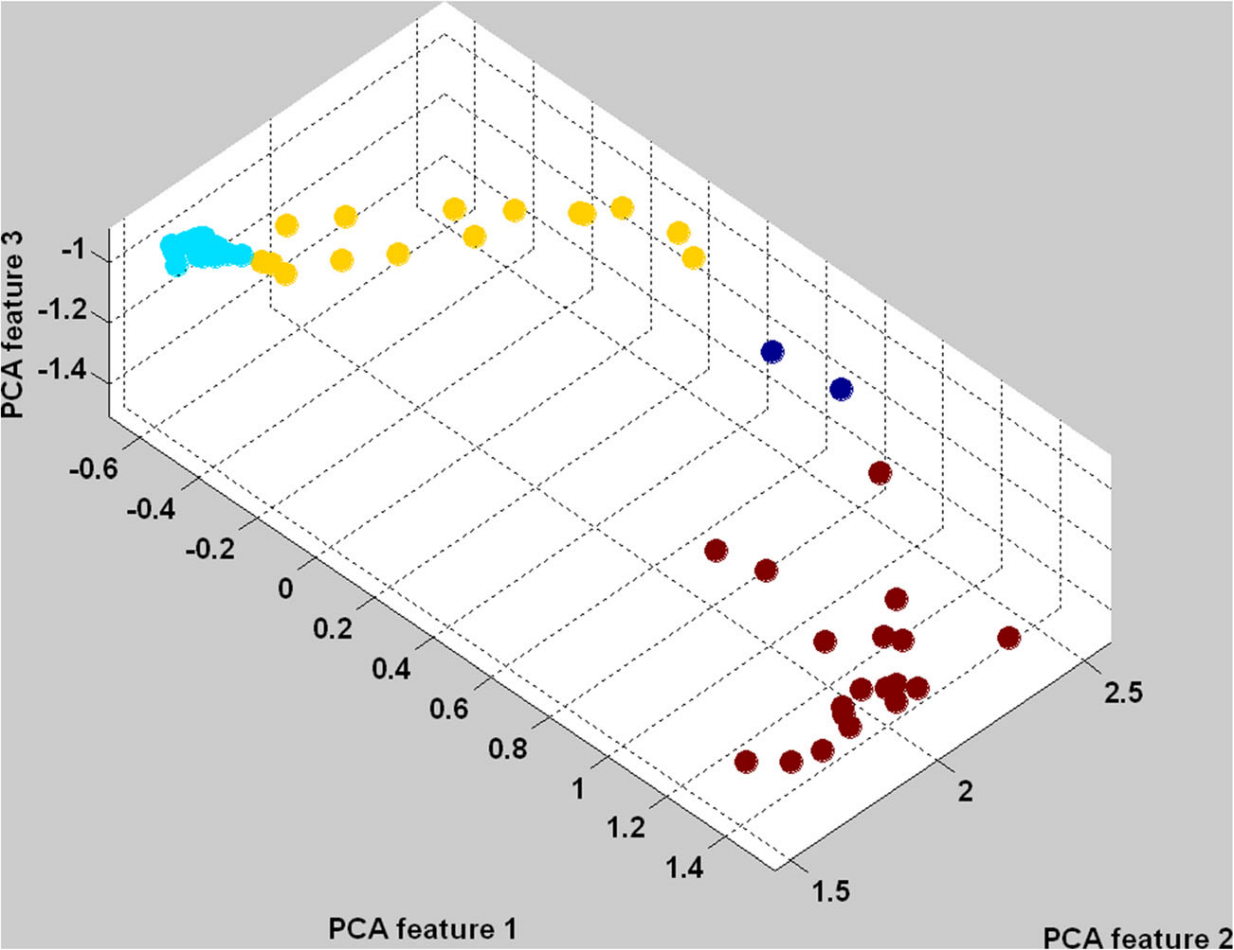
This figure presents three-dimensional projection of six-dimensional feature space (1) using principal component analysis. Each point represents a single MoCap frame with color-coded GDL key frame

The wearable body sensor enables to collect big data collections for which approaches know from other fields of big-data analysis can be applied [49, 50]. This property is also utilized in automatic training algorithm for GDL technology. While using GDL for a classification task the one of the most challenging aspect is designing of appropriate script that defines key frames of actions. To find those key frames automatically we often use Reverse-GDL (R-GDL) approach published in [25]. The R-GDL utilizes the fact that after transferring the original MoCap data to features space key frames can be detected with k-means clustering algorithm. This situation if visualized in Fig. 5 where data assigned to key frames is color coded with the same color pattern as in Fig. 4.

## Results

**Fig. 6:**
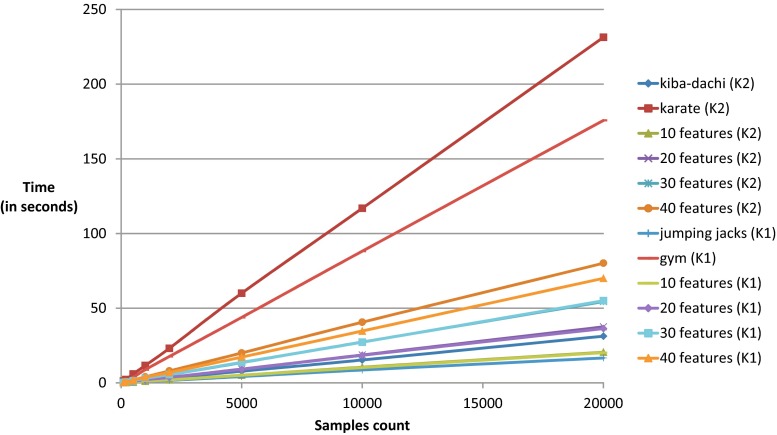
This figure visualizes data from Table 1

In third section we will evaluate the average performance of our adapted methodology. The implementation can be found on official website of GDL technology [51]. We are mostly interested in how much time is required to calculate features and to classify the input dataset. We have taken into account time of transferring data from hosting application to GDL engine, Lua scripts execution time and transferring data from GDL to host application. After this whole cycle the application obtains data that can be directly used by it. We have used 20-joints data with gym exercises recordings acquired with Kinect version 1 (K1), the same as we used in [25] and 25-joints data with karate techniques recordings acquired with Kinect version 2 (K2) [26]. The Lua scripts in which actions were defined varied in number of features definition and number of GDL instructions calls. The most basic scripts were 10- features, 20-features, 30-features and 40-features sets that were defined both for K1 and K2. All features were angles defined by vectors calculated from neighboring joint. The other scripts were codes that define kiba-dachi stand (about 4 kB of code), and definition of 12 karate actions (about 33 Kb of code) [30], jumping jacks exercise (4 kB) and 9 gym exercises (40 kB) [25]. We have used actions recordings consisted of 100, 200, 500, 1000, 2000, 5000, 10,000 and 20,000 frames. Evaluation was repeated 20-times for each Lua script and recording. The proposed method was evaluated on standard PC equipped with Intel Core i7-4770 CPU 3.40 GHz, 8 GB of RAM with Windows 7 Home Premium 64 Bit. The results in Table 1 and Fig. 6 are averaged results plus – minus standard deviation.

**Table 1:** This table presents averaged execution time (in milliseconds) plus-minus standard deviation of various Lua scripts that uses GDL implementation

	100	200	500	1000	2000	5000	10,000	20,000
kiba-dachi (K2)	171 ± 26	290 ± 35	754 ± 44	1683 ± 51	3062 ± 96	7824 ± 191	15,316 ± 278	31,249 ± 1218
karate (K2)	1173 ± 330	2225 ± 299	5913 ± 146	11,627 ± 358	23,114 ± 458	60,020 ± 788	116,892 ± 2052	231,344 ± 1046
10 features (K2)	125 ± 21	201 ± 22	505 ± 26	1123 ± 36	2064 ± 38	5116 ± 31	10,511 ± 377	20,566 ± 164
20 features (K2)	220 ± 21	366 ± 45	912 ± 43	2069 ± 56	3717 ± 57	9234 ± 55	18,677 ± 252	37,526 ± 915
30 features (K2)	301 ± 31	524 ± 65	1346 ± 69	2996 ± 83	5429 ± 83	13,550 ± 81	27,322 ± 374	54,419 ± 203
40 features (K2)	394 ± 35	778 ± 100	1977 ± 109	4010 ± 125	7999 ± 117	20,018 ± 140	40,615 ± 618	80,120 ± 500
jumping jacks (K1)	104 ± 16	166 ± 18	425 ± 25	1099 ± 149	1659 ± 21	4220 ± 73	8615 ± 409	16,684 ± 69
gym (K1)	722 ± 260	1251 ± 164	3710 ± 185	8806 ± 160	17,655 ± 221	43,832 ± 187	88,156 ± 646	175,715 ± 2004
10 features (K1)	121 ± 12	195 ± 22	498 ± 27	1148 ± 35	2024 ± 49	5087 ± 117	10,200 ± 288	20,211 ± 183
20 features (K1)	215 ± 25	359 ± 45	901 ± 50	2097 ± 63	3652 ± 52	9092 ± 38	18,439 ± 253	36,333 ± 38
30 features (K1)	309 ± 24	534 ± 67	1368 ± 65	3059 ± 100	5504 ± 85	13,744 ± 88	27,231 ± 500	55,012 ± 126
40 features (K1)	377 ± 31	671 ± 84	1710 ± 95	3828 ± 112	6921 ± 103	17,249 ± 115	34,723 ± 157	69,997 ± 448

Each row represents various features, action and actions groups that are evaluated for different number of motion capture frames (in columns)

**Fig. 7:**
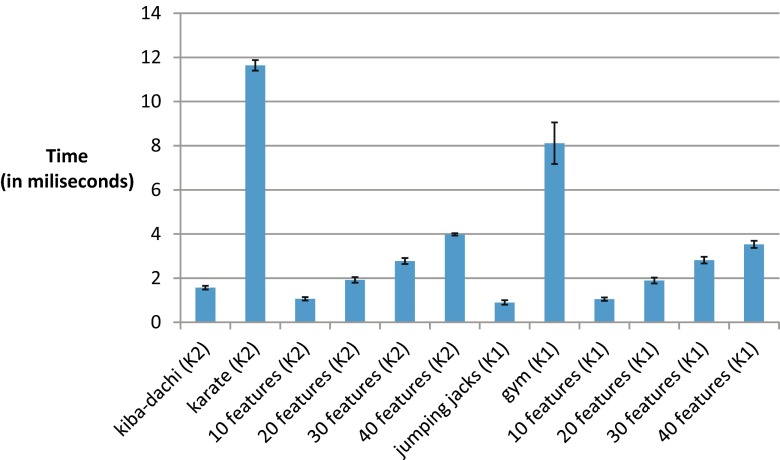
This figure visualizes data from Table 2

Basing on the obtained data in Table 2 and Fig. 7 we have shown what is an average execution time of single MoCap frame calculation plus – minus standard deviation.

**Table 2:** This table presents averaged execution time (in milliseconds) plus-minus standard deviation of various Lua scripts that uses GDL implementation for a single motion capture frame

Feature, action or action group name	Execution time (in milliseconds)
kiba-dachi (K2)	1.57 ± 0.08
karate (K2)	11.64 ± 0.24
10 features (K2)	1.06 ± 0.08
20 features (K2)	1.92 ± 0.13
30 features (K2)	2.77 ± 0.13
40 features (K2)	3.98 ± 0.05
jumping jacks (K1)	0.90 ± 0.10
gym (K1)	8.11 ± 0.94
10 features (K1)	1.05 ± 0.08
20 features (K1)	1.89 ± 0.13
30 features (K1)	2.82 ± 0.15
40 features (K1)	3.53 ± 0.16

## Discussion

The results presented in previous section show that processing time of Lua implementation of GDL methodology operates in fast and reliable way. As can be seen in Table 1 and Fig. 6 there is a nearly linear dependence between number of processed frames and time of processing. That proves that the method is stable and can operate without disturbance continuously. The test performed on features set for 10, 20, 30 and 40 features proves that there is no significant difference in processing time for 20 and 25 joints dataset. This is also quite natural that the larger the movement description is the more time is require to process the single MoCap frame. The execution time for 40-features K1 is 3.53 ± 0.16 milliseconds and for K2 is 3.98 ± 0.05 that means that it is possible to process MoCap dataset with frequency over 250 Hz which is sufficient for most up-to-date hardware of that type. The slowest processing was obtained for karate actions classification dataset that recognizes 12 different actions (11.64 ± 0.24 milliseconds per frame). That means that frequency of frame processing is about 85 Hz which is fast enough for signal classification task. Summing up the GDL methodology adapted to new functionalities satisfies needs of sport and rehabilitation data analysis and classification.

## Conclusions

In this paper we showed that Lua language can be successfully used for adaptation of the GDL classifier to new scientific tasks. The newly applied scripting language allows easily extension and integration of classifier with other software technologies and applications. As it was discussed in the previous section the obtained execution speed allows using the methodology in the real-time motion capture data processing where capturing frequency differs from 100 Hz to even 500 Hz depending on number of features or classes to be calculated and recognized. Due to this fact the proposed methodology can be used to the high-end motion capture system. We anticipate that using novel, efficient and effective method will highly help both sport trainers and physiotherapist in everyday tasks. For example pattern recognition and data mining methods can supply both sport and rehabilitation data evaluation. The proposed approach can be directly applied to MoCap data kinematics analysis (evaluation of motion without regard to the forces that cause that motion). However kinetics (the study of movements under the action of forces) will require additional data source beside MoCap for example ground reaction forces acquired by force plate or other force type collected with dynamometer. That additional data stream can be easily integrated with our methodology and the forces can be calculated without changing already established framework. However sole kinematics is sufficient for many applications in sport, medicine, physiotherapy and rehabilitation. Actions can be described using derivatives of displacement like velocity or acceleration. The ability to apply pattern recognition methods for GDL description can be utilized in virtual reality environment similarly to that described in [27, 52] and used for training or treatment.
